# Supplementary material for: β-Carotene Supplementation and Risk of Cardiovascular Disease: A Systematic Review and Meta-Analysis of Randomized Controlled Trials
Source: Nutrients. 2022 Mar 18;14(6):1284. doi: 10.3390/nu14061284 (PMC8950884; doi:10.3390/nu14061284)
Supplement: Supplementary file 1 [file nutrients-14-01284-s001.zip › nutrients-1606172-supplementary.pdf]

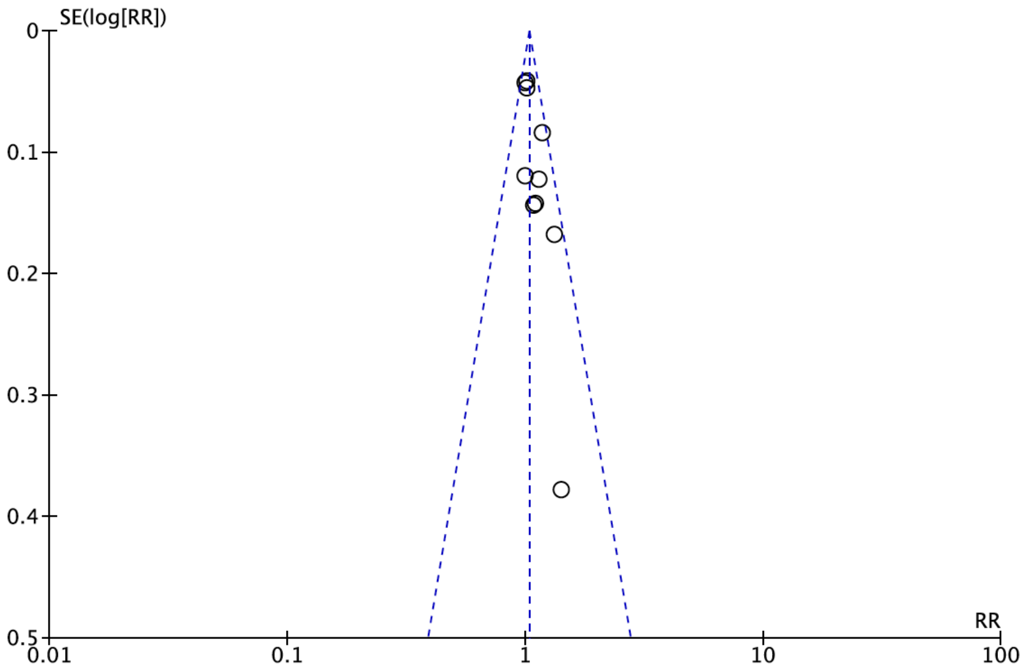

Figure S1. Major cardiovascular incidence funnel plot.

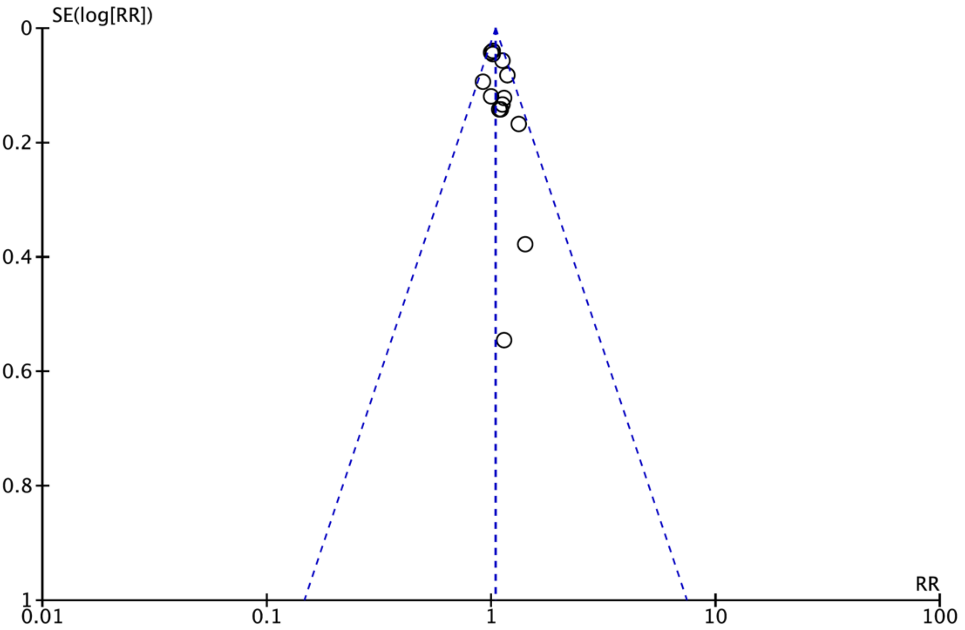

Figure S2. Overall cardiovascular incidence funnel plot.

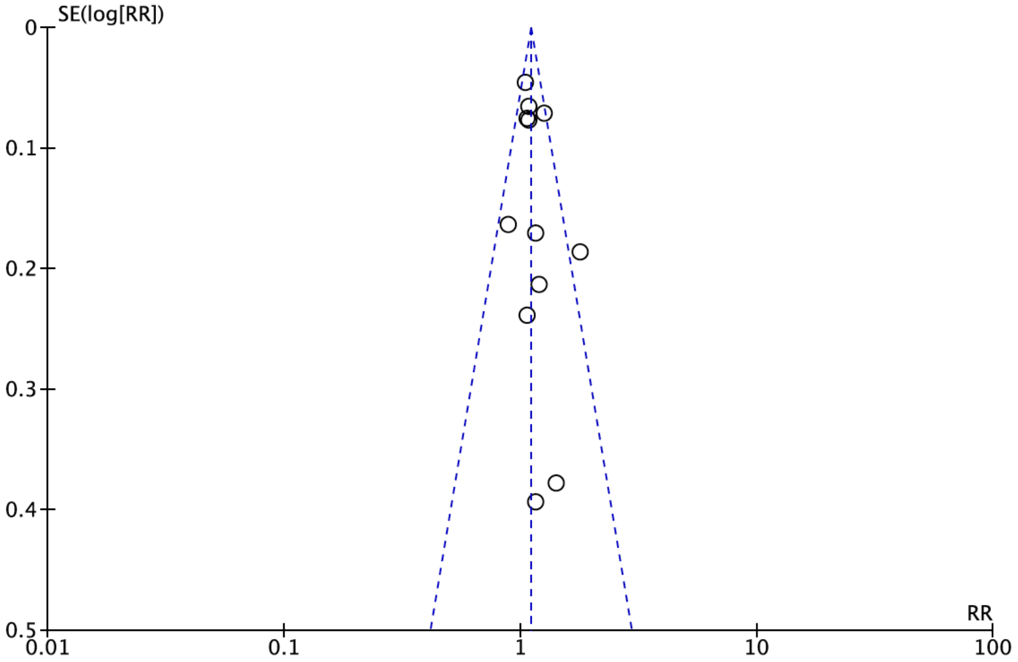

Figure S3. Cardiovascular mortality funnel plot.

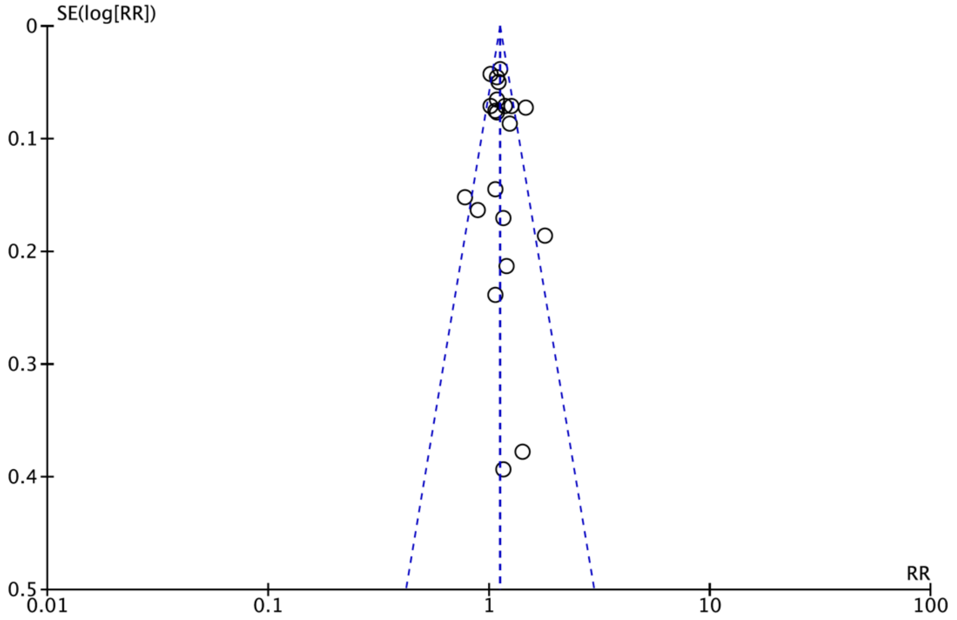

Figure S4. Overall mortality funnel plot.

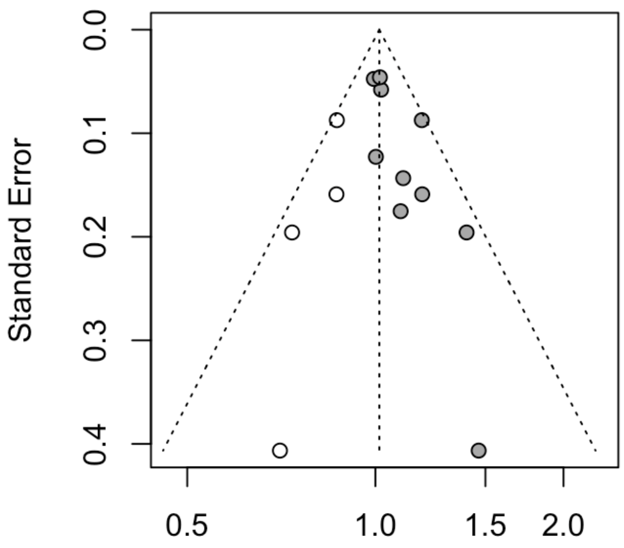

Figure S5. Trim-and-fill funnel plot for major cardiovascular disease incidence.
